# Knowledge Author: facilitating user-driven, domain content development to support clinical information extraction

**DOI:** 10.1186/s13326-016-0086-9

**Published:** 2016-06-23

**Authors:** William Scuba, Melissa Tharp, Danielle Mowery, Eugene Tseytlin, Yang Liu, Frank A. Drews, Wendy W. Chapman

**Affiliations:** Department of Biomedical Informatics, University of Utah, Salt Lake City, UT 84108 USA; Department of Biomedical Informatics, University of Pittsburgh, Pittsburgh, PA 15206 USA; University of California, San Diego, CA 92093 USA; Department of Psychology, University of Utah, Salt Lake City, UT 84108 USA

**Keywords:** Natural Language Processing, Information extraction, Semantics, Knowledge representation, Unified Medical Language System

## Abstract

**Background:**

Clinical Natural Language Processing (NLP) systems require a semantic schema comprised of domain-specific concepts, their lexical variants, and associated modifiers to accurately extract information from clinical texts. An NLP system leverages this schema to structure concepts and extract meaning from the free texts. In the clinical domain, creating a semantic schema typically requires input from both a domain expert, such as a clinician, and an NLP expert who will represent clinical concepts created from the clinician’s domain expertise into a computable format usable by an NLP system. The goal of this work is to develop a web-based tool, Knowledge Author, that bridges the gap between the clinical domain expert and the NLP system development by facilitating the development of domain content represented in a semantic schema for extracting information from clinical free-text.

**Results:**

Knowledge Author is a web-based, recommendation system that supports users in developing domain content necessary for clinical NLP applications. Knowledge Author’s schematic model leverages a set of semantic types derived from the Secondary Use Clinical Element Models and the Common Type System to allow the user to quickly create and modify domain-related concepts. Features such as collaborative development and providing domain content suggestions through the mapping of concepts to the Unified Medical Language System Metathesaurus database further supports the domain content creation process.

Two proof of concept studies were performed to evaluate the system’s performance. The first study evaluated Knowledge Author’s flexibility to create a broad range of concepts. A dataset of 115 concepts was created of which 87 (76 %) were able to be created using Knowledge Author. The second study evaluated the effectiveness of Knowledge Author’s output in an NLP system by extracting concepts and associated modifiers representing a clinical element, carotid stenosis, from 34 clinical free-text radiology reports using Knowledge Author and an NLP system, pyConText. Knowledge Author’s domain content produced high recall for concepts (targeted findings: 86 %) and varied recall for modifiers (*certainty*: 91 % *sidedness*: 80 %, *neurovascular anatomy*: 46 %).

**Conclusion:**

Knowledge Author can support clinical domain content development for information extraction by supporting semantic schema creation by domain experts.

**Electronic supplementary material:**

The online version of this article (doi:10.1186/s13326-016-0086-9) contains supplementary material, which is available to authorized users.

## Background

### Natural language processing

Natural Language Processing (NLP) provides a set of computational methods and techniques for automatically extracting and structuring information from free-text documents. NLP research has been successfully applied to free texts for several applications ranging from semantic search to information extraction to text analytics [1–3]. The development and availability of biomedical knowledge resources such as the Unified Medical Language System [4], has enabled biomedical NLP to move beyond retrieval and classification to modeling of semantic predicates represented in the literature [5]. Within the clinical domain, NLP systems have been implemented to support pharmaco-vigilance, patient screening, patient narrative summarization, and quality improvement [6–13]. The development of text processing pipelines and components specific to clinical text such as the Medical Language Extraction and Encoding System (MedLEE) [14], clinical Text Analysis and Knowledge Extraction System (cTAKES) [15], and Health Information Text Extraction [16] have permitted the analysis of clinical free texts e.g., emergency department notes, radiology reports, etc. using lexical, syntactic, and semantic information [17].

### Ontologies

NLP tools designed to support information extraction routinely use the Web Ontology Language (OWL) to provide a structured way to represent domain content [18–21]. In order for an NLP tool to use a given domain ontology, the tool must contain code to parse and interpret the data model represented in the ontology. This creates a close coupling between the ontology and the NLP tool. It is generally not possible to directly share the domain ontology used in one NLP tool with another NLP tool and semantic schematic changes are not easily propagated between tools. To help resolve this issue of incompatibility, a common type system [22] was developed which provides a common framework to create ontologies across a range of clinical domains. Our lab has converted the common type system described by Wu et al. into OWL format and extended its content using the Secondary Use Clinical Element Models (Secondary Use CEMs) [23]. We use this new OWL-base common type system, which we call the Schema Ontology, as the framework to create domain specific ontologies.

The Schema Ontology can be loaded into Protégé [24] or other OWL editors and used as the template for domain ontology creation. Domain ontology creation in this manner, however, requires deep understanding of OWL and an understanding of the structure of the Schema Ontology data model. This creates a potentially burdensome learning curve for those users who simply want to create Schema-Ontology-based domain ontologies and have little training in ontology development. To improve ease of use and support wide-spread adoption of the Schema Ontology, a system which minimizes complexity and allows simple interaction for users is needed. Knowledge Author provides a simple user interface to guide users in development of complex domain ontologies. Furthermore, a domain ontology development tool that supports collaborative editing and has built-in access to the UMLS would speed up the domain ontology creation process. Many OWL editors, such as Protégé or NeOn [25] allow user-created plugins to extend their functionality; however, there are no editors that are sufficiently modifiable to support all of this desired functionality. In this paper we introduce Knowledge Author which provides a web-based interface that is simple to use, facilitates domain content development with direct UMLS terminology lookup, and supports collaborative domain content creation.

## Implementation

### Terminology

The terminology used in the domains of clinical NLP and ontology creation can often vary; however, for the purposes of this paper the following terms are defined as such:

- *Semantic Schema* – the target extraction template for an NLP tool.
- *Atomic Concept* – a concept found in a standardized terminology such as the UMLS. For example – PNEUMONIA, TEMPERATURE, COUGH, or IBUPROFEN.
- *Lexical Variant* – a lexical variant is another way of phrasing an atomic concept or modifier in the clinical text. This can include synonyms, misspellings and abbreviations. For example – two lexical variants for TEMPERATURE are “temperature” and “temp”.
- M*odifier* – additional information that narrows down, or modifies an atomic concept. Knowledge Author separates the modifiers into two distinct types – shared and semantic. Shared modifiers are applicable to all concepts (with the exception of “Person” concepts which has its own unique set of modifiers such as age, race and gender). Semantic modifiers vary depending on the semantic type associated with the concept. For example, a concept with a semantic type of *Medication* will have a different set of available modifiers than a concept with semantic type *Vital Sign*.
- *Concept* – the combination of the atomic concept with its associated modifiers and their lexical variants. For example – 80 mg Ibuprofen is comprised of the atomic concept IBUPROFEN and semantic modifiers of *Dosage*: 80 mg. Lexical variants for IBUPROFEN can include “Advil”, “Midol”, “Motrin”, “Ibu”, and “Ibuprofen”, etc. Lexical variants for *Dosage* can include “80 mg”, “80 mg”, and “0.08 g”, etc.

### Knowledge Author overview

The overall goal of Knowledge Author is to aid a user in quickly creating a semantic schema, which is the target extraction template for a clinical NLP tool. The semantic schema represents salient concepts of interest to be extracted from the clinical text. It contains a list of atomic concepts, associated modifiers and the lexical variants for those concepts and modifiers. It is the job of the NLP system to extract words and phrases associated with these concepts and modifiers from the clinical text then map this information to the concepts in the semantic schema.

Knowledge Author provides a web-based graphical user interface that guides the user in developing a semantic schema, which is output as an OWL ontology. Knowledge Author standardizes the semantic schema creation process by constraining concept creation to a set of standard semantic types (e.g., Procedure, Medication) and by only allowing the user to assign a pre-defined set of modifiers to the concept. The semantic types and modifiers are based on the Secondary Use Clinical Element Models and the Common Type System (CTS). The Secondary Use CEMS are semantic types and modifier sets used for computerized provider entry and secondary use of clinical data, and the CTS are semantic type sets used for information extraction from clinical text. By adhering to a standardized data model it becomes possible to use the output of Knowledge Author in any NLP system which implements that model.

Knowledge Author also supports the semantic schema creation process by:

- Providing domain content suggestions through mapping of user-created concepts to concepts in the UMLS Metathesaurus database. This allows the automatic import of synonyms, concept definition, and semantic type into the Knowledge Author interface.
- Supporting modifier creation through the use of dropdown menus and the filtering of possible modifiers to only those relevant to a given concept type. Dropdown menus are possible because the Knowledge Author data model has a fixed set of allowable modifiers.
- Allowing the user to store and share their semantic schemas in an organized way.
- Supporting collaborative development of domain content.

### Using Knowledge Author

**Fig. 1:**
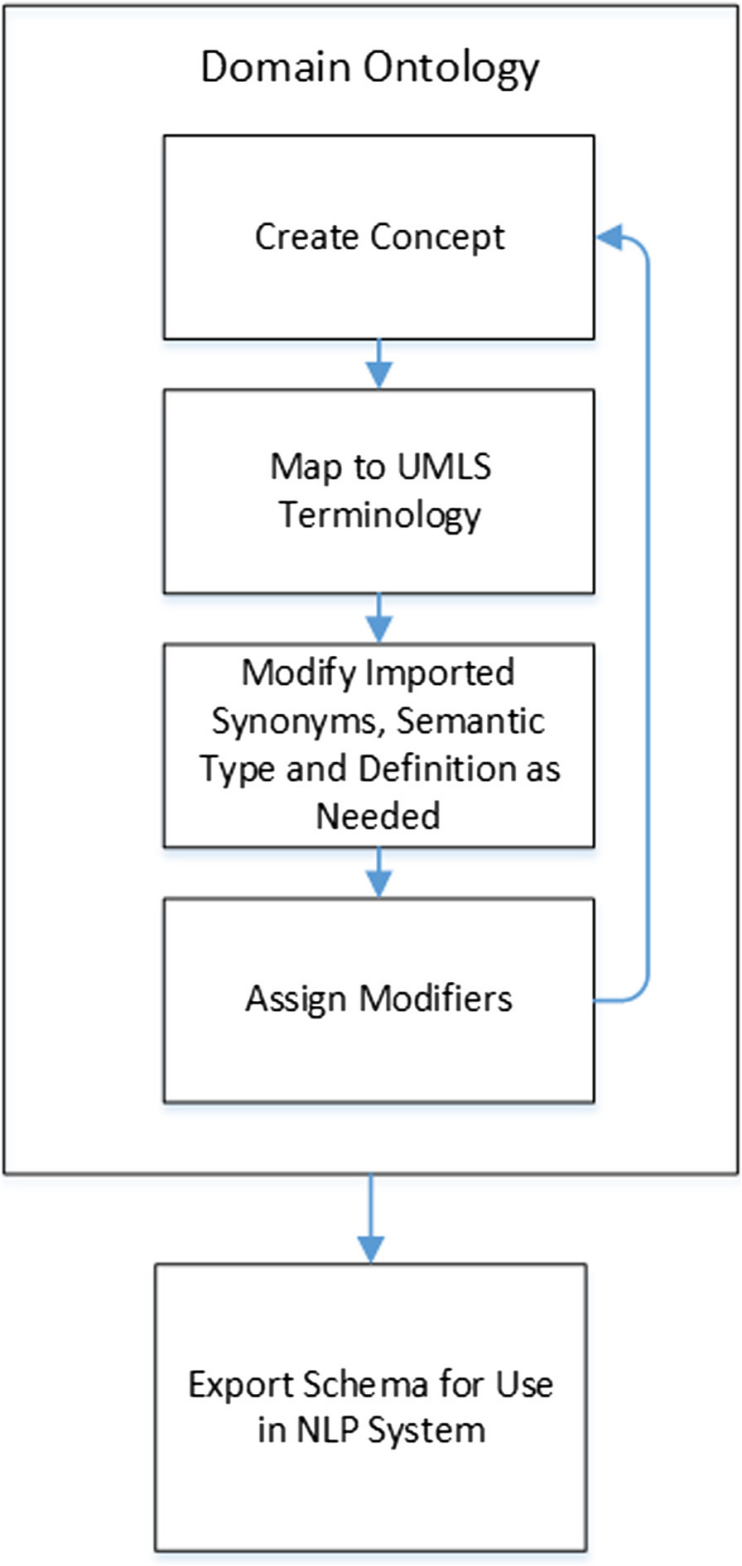
Illustrates the common set of steps to create a semantic schema using Knowledge Author. It is not required to map a concept to UMLS terminology as the synonyms, definition and semantic type can be entered in manually through the Knowledge Author interface

To illustrate the use of Knowledge Author, the creation of an example semantic schema for carotid stenosis will be walked through. Figure 1 illustrates the Knowledge Author workflow to be described below.

#### Defining a concept

The first step in Knowledge Author is to create a concept. Knowledge Author supports creation of two types of concepts: Person and Event. A Person concept can be defined with modifiers such as *birth date, death date, race, age,* and *gender* to facilitate creation of complex concepts such as African American females above 65 years of age.

**Fig. 2:**
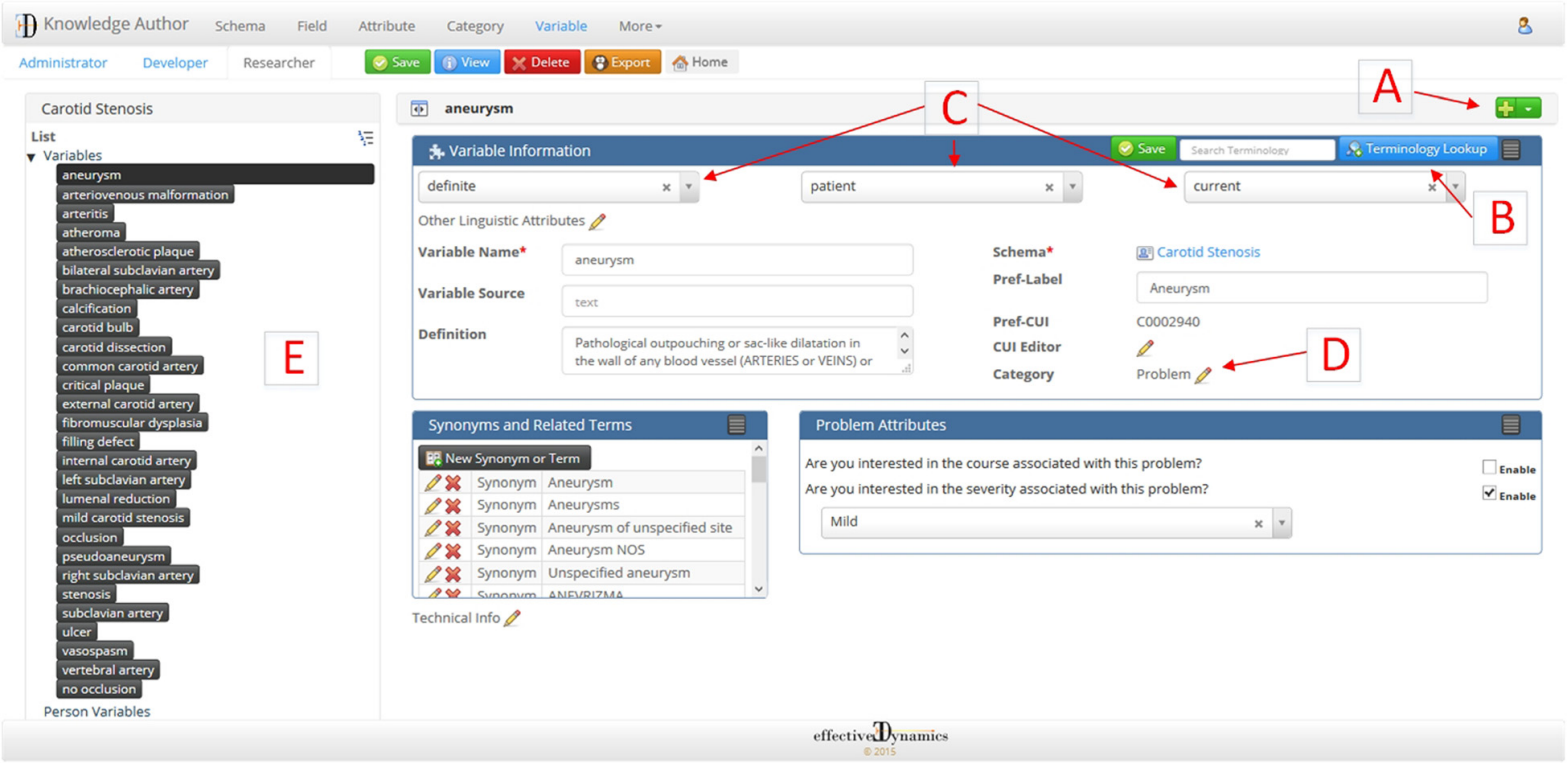
Knowledge Author concept creation interface. The large red letters with arrows point out **a**) concept creation button; **b**) terminology lookup button; **c**) shared modifiers; **d**) semantic type; **e**) concept list

**Fig. 3:**
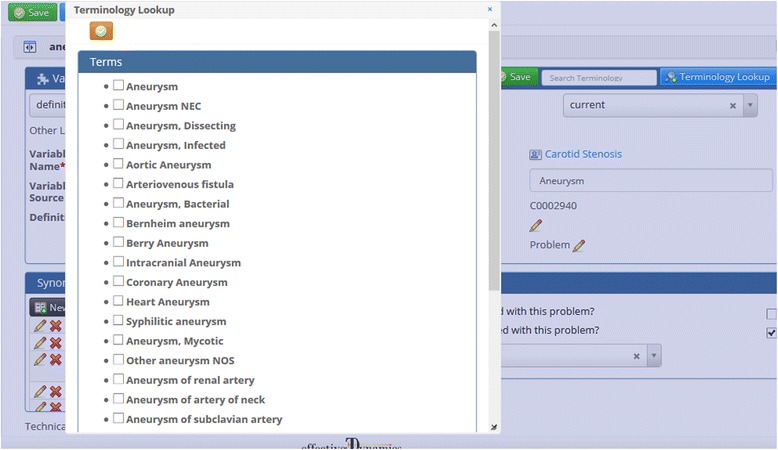
UMLS terminology lookup interface

The carotid stenosis use case only has Event concepts. To create the first concept – aneurysm – the new concept button “+” (Fig. 2a) was clicked and the concept name, “aneurysm”, was entered. Upon saving the new concept, the “Terminology Lookup” button (Fig. 2b) becomes available. Clicking that button allows the user to search the UMLS Metathesaurus for the concept name and displays a list of potential matches (Fig. 3). For this concept there is a UMLS atomic concept ANEURYSM which we choose. Knowledge Author will now download the definition, synonyms, semantic type and Concept Unique Identifier (CUI) for that atomic concept. All imported information can be changed, deleted, or supplemented as necessary. For the carotid stenosis example, twenty-six of the twenty-eight concepts were able to be mapped to UMLS concepts.

#### Choosing a semantic type

The next step is to assign a semantic type to the concept. If the concept is mapped to a UMLS atomic concept, the semantic type for the atomic concept will have already been downloaded and assigned to the concept (Fig. 2d). If not, the user can manually assign a semantic type. In the context of Knowledge Author, there are two types of modifiers – shared and semantic. The semantic type determines which type of semantic modifiers can be assigned to the concept.

#### Selecting semantic modifiers

Semantic modifiers are a type of modifier that is associated with specific semantic types. Each semantic type contains a number of possible semantic modifiers based on the Secondary Use CEMs and CTS. Each of the semantic modifiers has, in turn, a number of possible values associated with it. For example, the semantic type *Medication* allows the user to choose from semantic modifiers such as *dosage* or *delivery route.* The *delivery route* modifier has a number of possible values such as *oral* or *intravenous*. Table 1 lists the 12 semantic types, the modifier classes associated with each semantic type and the number of semantic modifiers associated with each modifier class.

**Table 1:** Semantic types, modifier classes, and modifiers available to the user

Semantic Type	Modifier Class	# of Modifiers	Sample of Modifiers
Allergy Intolerance	Allergy/Intolerance Type	2	allergy, intolerance
	Allergen	unlimited	any drug or food concept
	Severity	7	mild, moderate, severe
Anatomical Site	Body Side	3	right, left, bilateral
	Body Laterality	33	dorsal, medial, superior
Disease Disorder	Course	37	increased, worsened, maintained
	Severity	7	mild, moderate, severe
Encounter	From Location	unlimited	home, ER, SICU, nursing home
	To Location	unlimited	home, ER, SICU, nursing home
Lab/Test/Measurement	Abnormal Interpretation	3	abnormal, not abnormal, very abnormal
	Delta Flag	8	changed, unchanged, increased
	Lab/Test/Measurement Value	unlimited	500 cc, 100 kg, 12000 WBCs
	Ordinal Interpretation	35	excessive, high, low, positive
Medication	Medication Form	27	capsule, cream, liquid, tablet, pill
	Medication Route	21	inhalation, intradermal, oral
	Medication Strength	unlimited	500 mg
	Status Change	8	changed, unchanged, increased
	Dosage	unlimited	250 mg, 16 units
Patient Demographic	Birth Date	unlimited	
	Death Date	unlimited	
	Age	unlimited	
	Gender	2	
	First Name	unlimited	
	Last Name	unlimited	
	Middle Name	unlimited	
Problem	Course	37	increased, worsened, maintained
	Severity	7	mild, moderate, severe
Procedure Intervention	Delta Flag	8	changed, unchanged, increased
	Procedure Completion	3	complete, incomplete, N/A
	Procedure/Intervention Device	unlimited	
	Procedure/Intervention Method	unlimited	arthroscopic surgery
Sign or Symptom	Course	37	increased, worsened, maintained
	Severity	7	mild, moderate, severe
Social Risk Factor	Delta Flag	8	changed, unchanged, increased
	Social Risk Qualifier	6	occasional, frequent, social
	Social Risk Quantity	unlimited	5 packs, 3 drinks
	Social Risk Status	5	former risk, current risk
Vital Sign	Abnormal Interpretation	3	abnormal, not abnormal, very abnormal
	Delta Flag	8	changed, unchanged, increased
	Ordinal Interpretation	37	excessive, high, low, positive
	Vital Sign Value	unlimited	19 bpm, 86 %, 101.4 F

**Fig. 4:**
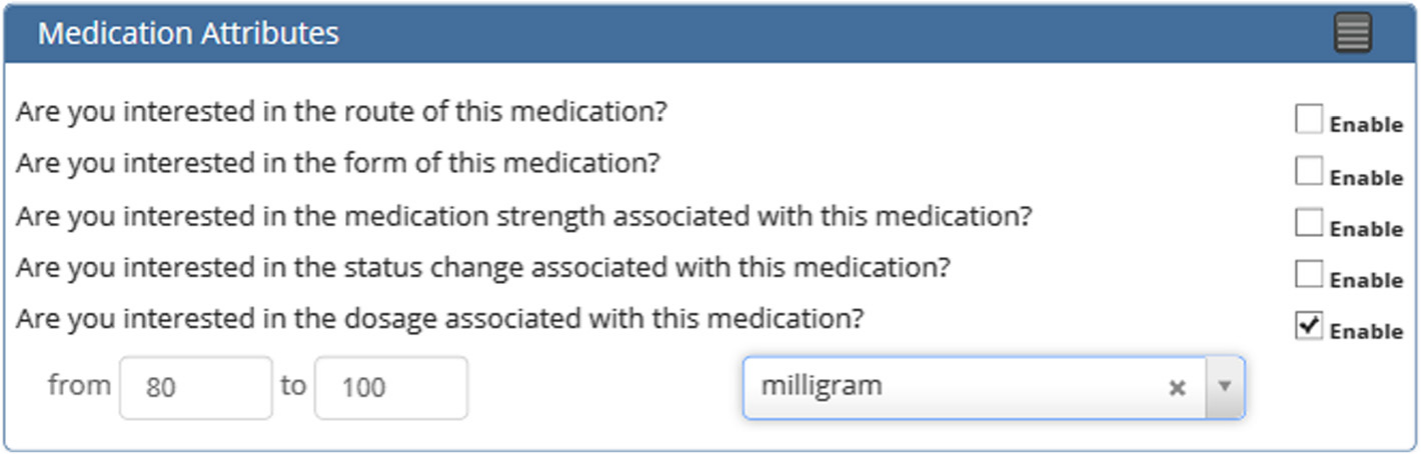
Semantic modifier interface box showing numeric range input boxes with units dropdown list

Semantic modifier values can either be chosen from a dropdown list, or for the case of numeric values, entered directly into an editable text box. Some modifiers, such as medication dosage, consist of two numeric value boxes and a dropdown list. The numeric value boxes allow the user to specify a value range, and the dropdown list is for units (Fig. 4). For example the user could create a concept for 80 to 100 mg Ibuprofen (Fig. 4). By leaving one or the other numeric value box empty concepts such as >80 mg Ibuprofen, or <80 mg Ibuprofen can be created. To create a single numeric value such as 80 mg Ibuprofen, enter the same number into both boxes. For the aneurysm concept created earlier, only the mild form is of interest so the sematic modifier of *severity* is enabled, and the value of *mild* is chosen from the dropdown list.

#### Selecting shared modifiers

**Fig. 5:**
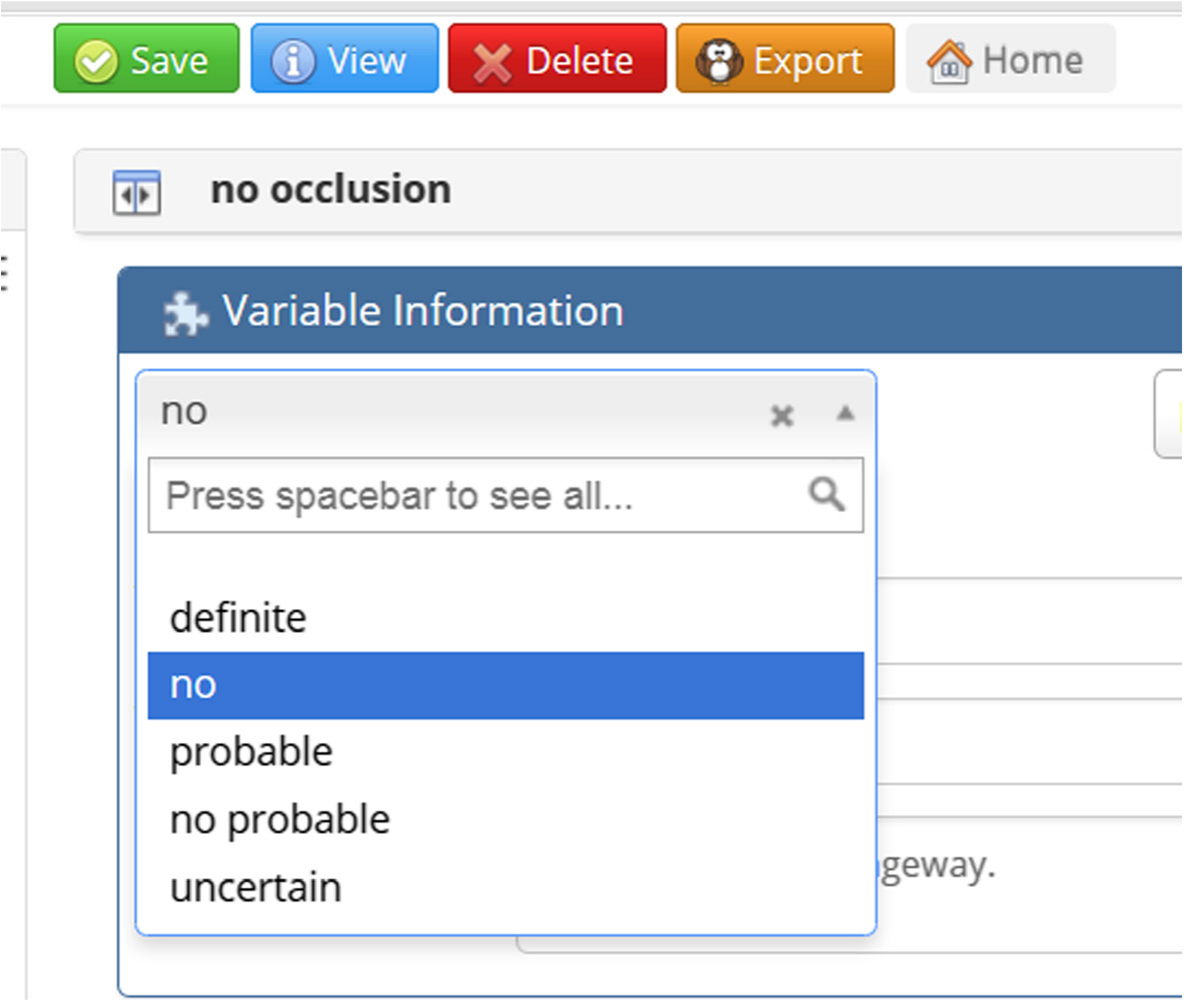
“Certainty” shared modifier dropdown list

A user can also narrow the definition of a concept through the use of shared modifiers. For all Event concepts, Knowledge Author allows the user to specify the *temporality* (whether the concept occurs in the past, present, or future), *certainty* (whether the concept is asserted, negated, or hedged), and *experiencer* (whether the patient or someone else experiences the concept) (Fig. 2c). Several other shared modifiers are also available (Table 2). For the carotid stenosis example, a concept for “no occlusion” is needed, so a new concept is created and linked to the atomic concept, OCCLUSION, which is then assigned the lexical variant for shared modifier for *certainty: no* from the certainty dropdown list (Fig. 5). The user could also use shared modifiers to create concepts such as family history of breast cancer or probable chest pain.

**Table 2:** Shared modifiers available to the user

Category	Shared Modifiers
Certainty	Definite Existence, Definite Negated Existence, Probable Existence, Probable Negated Existence
Experiencer	Patient, Family Member, Donor Family Member, Donor Other Member, Other Member
Temporality	Before, Before-Overlap, Overlap, After
Contextual Aspect	Continues, Initiates, Intermittent, Novel, Reinitiates, Terminates
Contextual Modality	Hypothetical, Conditional
Degree	Little, Most
Permanence	Finite, Permanent

#### Building a semantic schema

Once a concept is created and saved, the “+” button is clicked to create a new concept and the process described above is repeated. A concept, once created, is added to the concept list on the left hand side of the Knowledge Author GUI (Fig. 2e). The concept list can be arranged by the order in which the concepts were created, or by the semantic type they belong to. Once all of the concepts are created, the user can export the semantic schema for use in an NLP system.

### Exporting data

As the user works, Knowledge Author saves the user’s work to an internal database that is available upon login. Once all of the domain content is entered into Knowledge Author, the user can choose to export the data for use in an NLP system. The “Export” button at the top of the application will prompt the user to save the output file to their computer.

The file exported from Knowledge Author is OWL based and imports and uses the classes defined in the Schema Ontology file. This file contains the semantic categories and modifiers used by the interface as classes. The Schema Ontology is the base ontology file that organizes these classes into appropriate hierarchies. This Schema Ontology file is then imported into every new domain ontology created by Knowledge Author. During the export process, each of the concepts is exported as a subclass of the appropriate semantic category class (i.e., mild aneurysm is a subclass of the "Problem" class found in the Schema Ontology). All of the concept metadata (i.e., synonyms, misspellings, preferred term, CUI from UMLS, etc.) is added as annotation properties to that class. The modifiers are added as restrictions on the concept class (i.e., mild aneurysm has the restriction "hasSemAttribute some Mild_Severity"). Therefore, all of the data gathered by the Knowledge Author user interface is transformed into an ontological representation that can be parsed by a compatible NLP system.

It is also of note that the Knowledge Author output file can be viewed and modified directly by any OWL editor such as Protégé. This could be useful for users who want to use the Knowledge Author feature set, such as UMLS terminology mapping, semantic schema management, and dropdown lists, but have a small number of concepts with rare features that are not currently supported in Knowledge Author. Those concepts could be added by hand using the OWL editor.

### Collaborative development and semantic schema management

Over time a user can develop a large number of semantic schemas. Each schema a user creates is saved to the Knowledge Author database and is accessible to the user upon login. The five most recent schemas a user worked on are displayed in the quick launch window. All other schemas can be viewed in a searchable table.

A semantic schema can be designated by the creator as either “public” or “private”. Public schemas can be viewed and edited by anyone using Knowledge Author. This allows multiple users to work on the same schema. It also allows for the creation of a library of public schemas which can be used as the starting point for building a new schema in a similar domain. Private schemas can only be viewed and edited by the original creator.

### Software tools and specifications

Knowledge Author is a web-based platform written in Java 7 on top of a MySQL database. It runs on an Apache Tomcat 7 Server. The SeaCore [26] framework is used to facilitate the web development. The UMLS terminology is accessed through both the use of a local copy of the UMLS database and the Java based UMLS Terminology Service API 2.0 [27] which queries a remote UMLS Metathesaurus service. The mapping of a user’s concept to a UMLS atomic concept uses the UMLS Terminology Service API because of the complexity of performing that operation. The synonyms, definition, and semantic type for a concept are retrieved from the local copy of the UMLS for speed. The OWL API 3.4 [28] is used for converting the semantic schemas to OWL XML.

### Integration with existing NLP tools

Currently, only the pyConText [29] NLP system accepts the output from Knowledge Author as input. Work is also underway to integrate cTAKES and a developmental system called Moonstone [30] with the Knowledge Author output.

## Results and discussion

Knowledge Author standardizes the concept creation process by constraining the semantic types and modifiers that can be assigned to a concept to a discreet set. This enables the use of dropdown lists for assigning modifiers and allows for a standard output format which makes it possible to build NLP systems that use the output directly. We conducted two proof-of-concept studies, using different datasets, to assess the usability of Knowledge Author by demonstrating that (a) *the user interface is sufficiently flexible to allow for the creation of most concepts a user will want to create* and (b) *the output of Knowledge Author can be utilized by an NLP system to produce viable results.*

### User interface flexibility assessment

We assessed the flexibility of the Knowledge Author user interface by assembling a dataset of 115 concepts to be created using Knowledge Author. The Additional file 1 contains a full list of the concepts. The concepts were drawn from three disease or procedure areas: pneumonia, colonoscopy quality, and influenza. The concepts were selected to cover a range of complexity and provide a broad view of the types of concepts that can and cannot be created using Knowledge Author.

In order to assess whether or not the required concepts could be created using Knowledge Author, we considered three degrees of representation: *complete creation*, *partial creation,* and *no creation supported*.

We observed that 76 % (87 of 115) of the concepts for the pneumonia, colonoscopy, and influenza use cases could be *completely* created using Knowledge Author. Table 3 describes the 24 % (28 of 115) of concepts that could be *partially* created in their entirety (see Additional file 1 for a full list of 115 concepts created). Knowledge Author supported the creation of a very high proportion of “simple” concepts (69 of 73), but a lower proportion of “complex” concepts (18 of 42) by the knowledge engineer. Complex concepts include compound concepts developed from two semantic types, such as “lab test positive for influenza”. Knowledge Author supports creation of the concept “lab test positive” and “influenza” but does not yet support linking the two into a single concept. Knowledge Author, also, does not support creation of concept representing a single atomic concept with a set of modifiers combined with a disjunction, such as “new or progressive infiltrate”. The four “simple” concepts that were not able to be created in Knowledge Author are a result of the required modifiers not being listed in the Knowledge Author data model.

**Table 3:** Types and number of concepts that were not able to be created in Knowledge Author

Reason Not Created	Total # of Concepts	% of Total (115)
Element or modifier type not found in Schema Ontology	21	18 %
Relation between concepts missing - could only create separate concepts without linking	7	6 %

Even though Knowledge Author does not support the creation of some concepts, it is possible to add the desired data by hand outside of Knowledge Author. The Knowledge Author data model allows for the use of the Semantic Web Rule Language (SWRL) [31] rules, even though the Knowledge Author interface itself does not. SWRL is an OWL-based rule language. Through manual editing of the Knowledge Author output file, complex variables can be created by inserting SWRL rules. For modifiers that are not in the data model, it is possible to add the appropriate modifier classes by hand to the Knowledge Author output file. Correctly designed NLP tools that use the Knowledge Author output are able to handle user created classes. Having to add information outside of the Knowledge Author interface is time consuming and as Knowledge Author matures we expect to expand its functionality to cover the vast majority of concepts.

### Knowledge Author-powered information extraction evaluation

We assessed the viability of the Knowledge Author output for use in clinical NLP by creating a semantic schema for carotid stenosis in Knowledge Author and using it as the target extraction template in the pyConText [32, 33] NLP system.

pyConText is a regular-expression, rule-based information extraction system which accepts two files – one for target concepts and one for associated modifiers. The target file contains regular expressions or lexical variants describing target concepts of interest such as those representing carotid disease. The modifier file contains regular expressions or lexical variants describing the types of modifiers such as *certainty, anatomical location* or *temporality*. A software script was written to automatically marshal the data contained in the Knowledge Author output file into the file format and schema supported by pyConText.

We selected 34 carotid ultrasound reports from the MT Samples corpus [34] that were used in a previous study [32]. The reports were de-identified and selected at random from the MT Samples corpus. Two physicians independently annotated each report and adjudicated each disagreement with consensus review using an annotation tool called eHOST [35]. Each report was annotated for the targeted finding concepts for carotid stenosis along with the following associated modifiers: *certainty, sidedness,* and *neurovascular anatomy*.

We applied pyConText using the Knowledge Author semantic schema to the texts and converted its output to Knowtator.xml to be read into eHOST to conduct our error analysis. We computed recall for each type of target and modifiers (the proportion of concept mentions correctly identified from the reference standard) because we are predominately concerned with whether we have enough lexical variants to identify these concepts from free-text.

Reasonably high recall was achieved identifying targeted finding concepts (86 %) and shared modifiers (*certainty:* 91 %) and high to low recall for the semantic modifiers (*sidedness:* 80 %, *neurovascular anatomy*: 46 %) (Table 4).

**Table 4:** pyConText performance leveraging Knowledge Author knowledge base

Concept	Types	Total	Correct	Recall
Targets	Findings	79	68	86 %
*Modifiers*	*Certainty*	11	10	91 %
	*Sidedness*	41	33	80 %
	*Neurovascular Anatomy*	41	19	46 %

The low recall can be partially attributed to missing cues from the terminology lookup. In particular, many false negatives were due to missing acronyms and abbreviations in the semantic modifier file e.g., “ICA” which stands for “*neurovascular anatomy: Internal carotid artery*” and “l” which stands for “*sidedness: left*” which are commonly used in carotid ultrasound reports. Additionally, low recall can be partially attributed to the inability for Knowledge Author to represent ranges of severity for some semantic modifiers e.g., “70-80 %” which indicates significant stenosis. We are actively incorporating this functionality in the system. A manual input of additional acronyms and abbreviations using the Knowledge Author synonym interface and manual input of regular expressions for semantic modifiers using an OWL editor could improve the results. Overall, this result suggests that the Knowledge Author output has the potential to be used by an NLP system to create viable results.

### Future development

We are continuing to develop Knowledge Author and add new features. Some of the features that we expect to be added in the near future include:

- Adding constructs that will allow users to link concepts together using relationships (i.e. “ibuprofen treats pain”) and logical operators.
- Allowing the user to search a default corpus of de-identified medical records for phrases that would potentially be retrieved for the new concept. This would allow the user to test the accuracy of synonyms and numeric thresholds.
- Allowing the user to share and collaboratively work on an ontology with a select group of users.

Knowledge Author is the first part of a pipeline that will allow the user to create an NLP schema, annotate documents, process documents using various NLP systems, and analyze the results. We envision an end-to-end system that allows the user to rapidly build custom clinical text queries using a variety of NLP systems. We are actively developing a recommendation module within the pipeline that will suggest new lexical variants for concepts and modifiers from clinical text leveraging active learning methods to improve recall i.e., acronyms and abbreviations observed from development data in real-time. Currently, only the pyConText algorithm uses the output from Knowledge Author. Additional systems are under development.

## Conclusions

Knowledge Author is a new, web-based tool for building a semantic schema of domain content that could be used in an NLP application. It leverages three existing knowledge resources – the Secondary Use CEMs, CTS, and the UMLS – to provide the user with relevant information for creation of domain-specific concepts, which allows for rapid semantic schema creation. The output of Knowledge Author can be used directly as input into compatible NLP systems.

## Availability and requirements

Knowledge Author is publically available and can be found at http://blulab.chpc.utah.edu/KA/. The user can create an account to access the tool by clicking on the “Create Account” link. The data model used by Knowledge Author can be found at http://blulab.chpc.utah.edu/ontologies/SchemaOntology.owl. The completed carotid stenosis semantic schema can be found at http://blulab.chpc.utah.edu/ontologies/schemas/bscuba/carotid_stenosis.owl and in the Additional file 2.

## Abbreviations

cTAKES: clinical Text Analysis and Knowledge Extraction System; CTS: Common Type System; CUI: Concept Unique Identifier; eHOST: extensible Human Oracle Suite of Tools; HiTex: Health Information Text Extraction; MedLEE: Medical Language Extraction and Encoding System; NLP: Natural Language Processing; OWL: Web Ontology Language; Secondary Use CEM: Secondary Use Clinical Element Model; SWRL: Semantic Web Rule Language; UMLS: United Medical Language System; XML: Extensible Markup Language

## Additional files

Additional file 1: Full list of use case concepts. (XLSX 12 kb) Additional file 2: Carotid Stenosis OWL file. (OWL 232 kb)
